# ShrinkBayes: a versatile R-package for analysis of count-based sequencing data in complex study designs

**DOI:** 10.1186/1471-2105-15-116

**Published:** 2014-04-26

**Authors:** Mark A van de Wiel, Maarten Neerincx, Tineke E Buffart, Daoud Sie, Henk MW Verheul

**Affiliations:** 1 Department of Epidemiology and Biostatistics, VU University medical center, De Boelelaan 1117, 1081 HV Amsterdam, The Netherlands; 2 Department of Mathematics, VU University, De Boelelaan 1081a, 1081 HV Amsterdam, The Netherlands; 3 Department of Medical Oncology, VU University medical center, De Boelelaan 1117, 1081 HV Amsterdam, The Netherlands; 4 Department of Pathology, VU University medical center, De Boelelaan 1117, 1081 HV Amsterdam, The Netherlands

**Keywords:** Differential expression, Shrinkage, Sequencing, Bayesian analysis

## Abstract

**Background:**

Complex designs are common in (observational) clinical studies. Sequencing data for such studies are produced more and more often, implying challenges for the analysis, such as excess of zeros, presence of random effects and multi-parameter inference. Moreover, when sample sizes are small, inference is likely to be too liberal when, in a Bayesian setting, applying a non-appropriate prior or to lack power when not carefully borrowing information across features.

**Results:**

We show on microRNA sequencing data from a clinical cancer study how our software ShrinkBayes tackles the aforementioned challenges. In addition, we illustrate its comparatively good performance on multi-parameter inference for groups using a data-based simulation. Finally, in the small sample size setting, we demonstrate its high power and improved FDR estimation by use of Gaussian mixture priors that include a point mass.

**Conclusion:**

ShrinkBayes is a versatile software package for the analysis of count-based sequencing data, which is particularly useful for studies with small sample sizes or complex designs.

## Background

Following the surge of count-based sequencing data, a plethora of software packages for differential expression analysis of such data has emerged [1]. Many of these methods are limited in use due to restrictions on the study design, the model and inference like a) 2- or *K *- group comparisons only; b) no random effects; c) no explicit solution for excess of zeros and d) no multi-parameter inference. We introduced ShrinkBayes as a versatile analysis method which allows generalized linear mixed models and zero-inflation and with, due to its multi-parameter shrinkage options, good reproducibility and power characteristics [2]. This paper illustrates the R-package ShrinkBayes on a challenging microRNA sequencing (miRseq) colon tumor-plus-metastasis study. In addition, we automated the use of mixture priors containing a spike, leading to improved FDR-based inference. Finally, we extend the class of admitted priors with mixtures of a multivariate point mass and a Gaussian product density to allow for powerful multi-parameter inference.

## Implementation

### Shrinkage

ShrinkBayes applies Integrated Nested Laplace Approximation, INLA[3], in combination with Empirical Bayes principles to provide shrunken parameter estimates and inference. In a Bayesian setting, multi-parameter shrinkage is effectuated by estimating hyper-parameters of priors. The core of ShrinkBayes is iterative estimation of priors: each prior is fit to the point-wise empirical mean of the *marginal* posteriors of those parameters *θ*_*i*_,*i *= 1,…,*p *= # features, that correspond to the prior [2]. Shrinkage is known to be potentially beneficial for dispersion parameters, but may be as important for parameters of interest to accomplish better inference [2] and for nuisance parameters to reduce their impact when unimportant [4].

A typical ShrinkBayes analysis consists of the following modules: a) Iterative Empirical Bayes estimation of multiple priors which need to obey the parametric forms included in INLA; b) Fitting of the full model and the null model; c) Updating one prior resulting from a) to a non-parametric or mixture prior to allow for for more flexibility and/or better inferential properties; d) Updating the posteriors of the corresponding parameters; e) Computing summary statistics including estimates of lfdr and (B)FDR. The steps are detailed in the Example section. Below we discuss novel implementations and methods with respect to [2].

### Setting

The setting is a generalized linear model. Let *j *= 1,…,*n *denote independent samples, *Y*_*ij *_be the data for feature *i *and sample *j*, *F *be the likelihood model (e.g. (zero-inflated) negative binomial) with mean *μ*_*ij *_and hyper-parameters ***γ***_*i *_and *g *() a link-function. Here, ***γ***_*i *_contains distribution parameters that are not linked to covariates, e.g. zero-inflation and over-dispersion. Then,

(1) $$\begin{array}{ll} Y_{\text{ij}} & {=}^{d}F(\mu_{\text{ij}},\gamma_{i})\\ g(\mu_{\text{ij}}) & =X_{j}^{\alpha }\alpha_{i}+X_{j}^{\beta }\beta_{i}, \end{array}$$

where ***β***_*i *_= (*β*_*i*1_,…,*β*_*iK *_) denotes the parameter(s) for which (joint) inference is desired, while ***α***_*i *_contains all the other regression parameters, including the intercept. In addition, $X_{j}^{\alpha }$ ($X_{j}^{\beta }$) denotes the *j*th row of the design matrix restricted to those columns of this matrix that are relevant for ***α***_*i *_(***β***_*i*_).

### Priors

ShrinkBayes inherits much of its flexibility from the INLA R-package, including its ability to deal with arbitrary designs and random effects. INLA, however, requires use of specific parametric priors. Since the prior may be crucial for inference in a multiple testing setting, we extended the class of admissible priors to non-parametric and parametric mixture priors [2].

ShrinkBayes was praised for its power and versatility, but also criticized for its poor FDR estimation in case of a point null-hypothesis for one parameter (so ***β***_*i *_= *β*_*i*_), *H*_0 *i *_: *β*_*i *_= 0 against *H*_1 *i *_: *β*_*i *_≠ 0 [1]. Here, we resolve this issue. In [1], a smooth non-parametric prior was used for *β*_*i*_, which does not suit *H *_0 *i*_. To promote more suitable priors, we simplified application of parametric mixture priors with a spike on zero by automating multi-grid parameter estimation of such priors, and increased their flexibility by allowing non-equal mixture proportions for negative and positive effects. Moreover, we implemented a mixture of a spike and a smooth non-parametric component (SpNP prior). For the Results, we focus on the Spike-Gauss-Gauss (SPGG) and SpNP priors:

(2) $$\begin{array}{lll} \text{SpGG} & =p_{0}\delta (0)+p_{-1}N(-\mu ,\tau^{2})+p_{1}N(\mu ,\tau^{2})\; & \\ & \;\text{ Spike-Gauss-Gauss’}\; \end{array}$$

(3) $$\begin{array}{lll} \text{SpNP} & =p_{0}\delta (0)+(1-p_{0})F_{\text{NP}}\; & \\ & \;\text{ Spike-Nonparametric’},\; \end{array}$$

where *δ *() is the dirac delta function, i.e. a spike. The spike is essential, because it allows the posteriors to have non-zero mass on the null-hypothesis, *β*_*i *_= 0, hence accommodating selection. The smooth parts of both these priors allow asymmetry between under and overexpression. All parameters are determined by maximizing the total (log-) marginal likelihood (i.e. the sum of marginal likelihoods over all features). This maximization is explicit for the parametric SpGG prior, whereas *F*_NP_ is obtained by the iterative marginal procedure [2] with the restriction that it contains maximally one mode on both the negative and positive half-plane. The restriction helps to identify *F*_NP _together with *p*_0_. In words, given a current proposal for *p*_0 _and *F*_NP _the iterative procedure proposes a new estimate of *p*_0 _and *F*_NP _by fitting the SpNP prior to the point-wise empirical mean (over features *i*=1,…,*p*) of the current posteriors *π*(*β*_*i*_|**Y**_*i*_), where the fit needs to respect the aforementioned restriction. Any reasonable starting value of *p*_0 _(we use 0.8) and *F*_NP _(we use a sufficiently vague central Gaussian, e.g. *N*(0,5)) can be used and convergence is checked by assessing the total (log-)marginal likelihood.

ShrinkBayes allows for other parametric priors, such as the  Spike-Gauss’ (SpG) and the  Spike-and-Slab’ (SpSlab). Both are mixtures of a point mass and a central Gaussian distribution, but the first has a data-adaptive variance fitted with the same direct maximization procedure as for the SpGG prior, whereas the latter has a prescribed large variance. Both alternatives are discussed in more detail in the Additional file 1.

### Multi-parameter inference

Multi-parameter inference is desirable when the parameters represent multiple groups or covariates with a similar interpretation. In a frequentist setting, this is often done by likelihood-ratio tests. Below we discuss the Bayesian counterpart. Suppose one aims at testing *H*_0*i *_: ***β***_*i *_= **0 **against *H*_1 *i *_: ***β***_*i *_≠ **0 **in a linear model *M *(***β***_*i*_), which also includes response **Y**_*i*_, covariates **X **and, possibly, additional parameters ***λ***_*i*_. Refer to the full model ${ℳ}_{1}=M(\beta_{i})$ when ***β***_*i *_is unconstrained and the null model ${ℳ}_{0}=M(0)$. Traditionally, comparison of two models is done by computation of the Bayes Factor (BF). However, in a multiple testing setting a good threshold for BF requires knowing *p*_0_, the proportion of true null models (see [5], Ch. 5). Then, thresholding for BF is directly linked to local fdr, which simply equals

(4) $$\begin{array}{ll} \text{lfdr} & =\pi_{0}=P({ℳ}_{0}|Y_{i})\\ & =\frac{p_{0}\text{ML}(Y_{i};{ℳ}_{0})}{p_{0}\text{ML}(Y_{i};{ℳ}_{0})+(1-p_{0})\text{ML}(Y_{i};{ℳ}_{1})}, \end{array}$$

where $\text{ML}(Y_{i};{ℳ}_{0})$ and $\text{ML}(Y_{i};{ℳ}_{1})$ are the marginal likelihoods under ${ℳ}_{0}$ and ${ℳ}_{1}$, respectively. On its turn, lfdr determines BFDR(*t*,**Y**_*i*_) = *E*[lfdr|lfdr < *t*] : the mean of all local fdrs smaller than *t*. Given its analogous interpretation to ordinary FDR [6] we prefer to define threshold *t *using BFDR(*t*,**Y**_*i*_) rather than lfdr. In any case, we need to compute $\text{ML}(Y_{i};{ℳ}_{0}),\text{ML}(Y_{i};{ℳ}_{1})$ and *p*_0_.

The marginal likelihoods $\text{ML}(Y_{i};{ℳ}_{0})$ and $\text{ML}(Y_{i};{ℳ}_{1})$ are conveniently supplied by INLA from the two separate fits of the models ${ℳ}_{0}$ and ${ℳ}_{1}$. Finally, *p*_0 _is determined by our *iterative joint procedure*[2], which determines the value of *p*_0 _(along with other parameters) that maximizes the total (log-)marginal likelihood with respect to prior:

(5) $$p(\beta_{i})=p_{0}\delta (\beta_{i}=0)+(1-p_{0})\overset{K}{\underset{k=1}{\prod }}N(0,\sigma_{k};\beta_{\text{ik}}),$$

hence a mixture of a multivariate point-mass (*δ*(***β *** = **0**)) and a Gaussian product density for the regression parameters ***β***_*i *_= (*β*_*i *1_,…,*β*_*iK *_). In particular when the true *p*_0_ is large, the total (log-)marginal likelihood may contain ridges and/or multiple modalities with respect to the parameters of (5). For example, when the true *p*_0 _is large a prior (5) with *small*${\hat{p}}_{0}$ and small values of *σ*_*k *_may also fit rather well. To counter this, we use the constraint *p*_0 _≥ 0.5 (which is realistic in most cases) and use a large default starting value of *p*_0 _(0.8). Moreover, iteration is stopped when the total (log-)marginal likelihood decreases by less than 0.1% to avoid  walking on a ridge’.

### Additional changes

In addition to the improved implementation of spike-priors and the multi-parameter inference, ShrinkBayes versions 2.3 and higher contain a number of novelties and changes compared to version 1.6, which corresponds to [2]. In particular, it is faster, because convergence of the parameters of the prior(s) is assessed in terms of total marginal likelihood instead of on the separate parameters. The new version also allows to approximate marginal likelihood for a null model from the results of the full model using the Savage-Dickey approximation [7]. This is particulary convenient for contrasts for which a null-model can not be defined without the use of constraints. Additional file 1, Section 2, contains more details and a full list of changes.

## Results

### Priors

To study which of the priors performs best in terms of FDR estimation and power, we compared them on simulated data sets, including those in [1].

#### ***Results on simulations for various effect size distributions***

The true effect size distribution, i.e. the true generating distribution of the parameter of interest, may have impact on what prior performs best. Hence, we study several effect size distributions, including a Gamma, *t*, Uniform and Gaussian mixture (see Additional file 1, Section 1). We compared performance of the SpGG, SpNP, SpG and SpSlab priors in terms of accuracy of FDR estimation, area-under-the-curve (AUC), number of detections and absence of detections when *H*_0*i *_is true for all features (*p*_0 _= 1). From the results (Additional file 1, Section 1) we conclude that SpGG and SpNP lead to accurate estimates of FDR and are very competitive in terms of power, whereas SpSlab is often too conservative; SpG generally performs well except for the (asymmetric) Gamma distribution for which it is less powerful than SpGG and SpNP. In the case *p*_0 _= 1, none of the prior returns a significant result at BFDR≤0.1, but the SpGG prior performs best in the sense that it produces the highest BFDRs.

#### ***Results on simulations in *** [1]

Next, we report results of ShrinkBayes with the SpGG and SpNP priors on simulations in [1], which compared several methods, including ShrinkBayes (referred to as ShrinkSeq), on a variety of data sets. ShrinkBayes was used with a smooth non-parametric prior (NP), so not containing a spike. The number of features equals 12500. We focus on data sets where counts are exclusively generated from the negative binomial. Moreover, we report results on the symmetric cases (in terms of up- and down-regulation) only (${\text{B}}_{2000}^{2000}$, *p*_0 _= 0.64 and ${\text{B}}_{625}^{625}$, *p*_0 _= 0.9), because for the asymmetric cases the normalization procedure used in [1] introduces artificial differential signal for the non-differential features. We do include a case with outliers which contains, on average, 5% outliers for 10% of the features (${\text{S}}_{625}^{625}$). For sample sizes we focus on *n *= *N*/2 = 5,10, because the ShrinkBayes results reported in [1] were relatively worse for those sample sizes.

Table 1 contains the results on FDR estimation. Note that the target FDR equals 0.05 here, in order to be consistent with [1]. We observe that ShrinkBayes with SpGG or SpNP is still liberal, but the results are much better than those for the NP prior. In fact, when comparing the results of Table 1 with those in Figure four of [1], we observe that ShrinkBayes has improved from the worse to at least average in terms of FDR estimation. In particular, for the data sets with outliers it outperforms 5/6 (4/6) [ *n *= 5(10)] of the other methods that are based on count distributions.

**Table 1:** FDR results for target FDR=0.05

**Data set**	***n *** **= *N */2**	**SpGG**	**SpNP**	**NP**^**∗**^
${\text{B}}_{2000}^{2000}$	5	0.085	0.078	0.29
${\text{B}}_{2000}^{2000}$	10	0.079	0.071	0.29
${\text{B}}_{625}^{625}$	5	0.115	0.115	0.37
${\text{B}}_{625}^{625}$	10	0.083	0.081	0.38
${\text{S}}_{625}^{625}$	5	0.111	0.108	0.38
${\text{S}}_{625}^{625}$	10	0.119	0.117	0.40

^∗^: as reported in [1].

Table 2 contains the results on AUC. Again, we observe a uniform improvement when using ShrinkBayes with SpGG or SpNP instead of NP. Strikingly, ShrinkBayes with both SpGG and SpNP generally outperforms all the other methods reported in [1] when it comes to AUC.

**Table 2:** Area-under-the-curves

**Data set**	***n*** **= *N */2**	**SpGG**	**SpNP**	**NP**^**∗**^	**Best**^**∗**^
${\text{B}}_{2000}^{2000}$	5	0.897	0.898	0.85	0.87
${\text{B}}_{2000}^{2000}$	10	0.949	0.951	0.91	0.93
${\text{B}}_{625}^{625}$	5	0.874	0.879	0.82	0.87
${\text{B}}_{625}^{625}$	10	0.937	0.940	0.88	0.93
${\text{S}}_{625}^{625}$	5	0.866	0.871	0.81	0.85
${\text{S}}_{625}^{625}$	10	0.923	0.927	0.87	0.92

^∗^: as reported in [1]. Best^∗^: Highest AUC of all other methods reported in [1].

### Multi-parameter inference: data-based simulation

We compare our solution for multi-parameter inference to the likelihood-ratio tests that are implemented in the popular RNAseq data analysis programs edgeR[8] and DESeq[9]. We believe such a comparison is most meaningful and fair when the data is simulated in a relevant and realistic way, preferably avoiding distributional assumptions as much as possible. Therefore, we generated the data in three steps. First, we create a realistic null data set: we simply re-sample 3*5  observations’ from our miRseq data set, independently for each of the 2060 features. Hence, per feature 5 observations are generated from the same empirical distribution for each of the 3 groups. Next, modest filtering on the number of non-zeros is applied, because this is recommended for the use of edgeR and DESeq: at least 3 non-zeros should be present. Finally, we need a realistic effect size distribution for the features. To avoid parametric assumptions this is estimated by *F*_NP_, the smooth component of the SpNP prior (3), for the groups in the miRseq study (organs). We create 20% differential features by sampling independently from *F*_NP_ for groups 2 and 3 and multiplying the respective counts by the exponentiated sampled effect sizes. This entire simulation was repeated 10 times.

**Figure 1:**
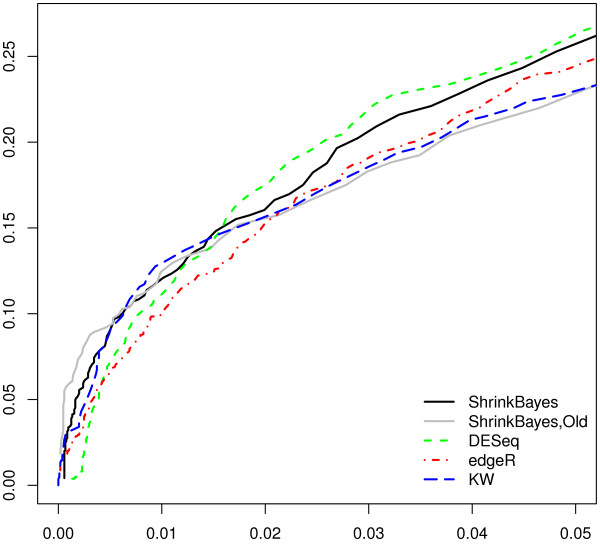
ROC curves for multi-parameter inference: mean False Positive Rate (FPR; x-axis) vs mean True Positive Rate (TPR; y-axis), as averaged over 10 repeats of the data-based simulation, which consists of 3 groups with 5 counts for ≈2000 features.

We analyzed the simulated data sets using ShrinkBayes, edgeR, DESeq and a simple nonparametric Kruskal-Wallis test. In addition, the old version of ShrinkBayes was applied with a smooth nonparametric prior and an *a posteriori* multiple comparison of the 3 groups, as suggested in [2]. Figure 1 shows the ROC curves, as averaged over the 10 repeats, for False Positive Rate (FPR) smaller than 0.05. We focus on this FPR range, because when using $\hat{\text{FDR}}\leq 0.1$ as a selection criterion, all 5 methods produce sets of significant features with FPR≤0.05. ShrinkBayes seems somewhat superior to edgeR across the entire range, while it is competitive with DESeq. Possibly due to the smoothness of the prior ShrinkBayes,Old performs a little bit better in terms of ranking than ShrinkBayes for very small FPR, but becomes inferior for larger values. The latter may be caused by loss of power when using a multiple comparison approach in a *K*-group setting. Surprisingly, the Kruskal-Wallis test seems to be very competitive, although it also loses power for larger values of the FPR.

ROC curves, however, only allow comparison of the rankings. In practice, the actual selection is most important. Table 3 shows the results summarized over the 10 repeats when using $\hat{\text{FDR}}\leq 0.1$ as selection criterion. Note that for all p-value-based methods we use the Benjamini-Hochberg FDR correction, which is appropriate here given the independent sampling per feature in our simulated data set. BFDR is used as an estimate of FDR in the ShrinkBayes setting. True FDR is evaluated on the selected sets by simply dividing the number of false positives by the total number of positives. Here, the differences are much clearer: the Kruskal-Wallis test is useless in this setting, because it does not select anything. ShrinkBayes,Old selects too much at a too high true FDR, probably due to the smooth prior, as discussed before. DESeq and ShrinkBayes produce better true FDRs (with the DESeq ones more variable), but, on average, ShrinkBayes detects almost four times as many features. edgeR selects more, but is both more liberal and more variable. In fact, as can be inferred from the ROC curves, ShrinkBayes would achieve a smaller true FDR with the same number of detections as edgeR.

**Table 3:** Number of detections (mean and standard deviation) at target FDR = 0.1 and true FDR for the set of detections (median and IQR: interquartile range)

**Method**	**# Detections**	**True FDR**
	**mean (sd)**	**Median (IQR)**
ShrinkBayes	37.4 (4.60)	0.171 (0.072)
ShrinkBayes, Old	132.1 (15.3)	0.509 (0.038)
edgeR	58.8 (12.9)	0.258 (0.120)
DESeq	10.4 (3.75)	0.191 (0.178)
Kruskal-Wallis	0 (0)	NA

Results are summaries from 10 repeats of the simulated data sets.

Note that ShrinkBayes is still liberal in the sense that it underestimates true FDR. This is probably due to the data not being generated from a specific parametric distribution. In particular, we observed that the data contains outliers for some features. Dedicated detection of such outliers can certainly reduce the number of false positives. A simple, heuristic, practical alternative is to additionally require for selection the corresponding *uncorrected *Kruskal-Wallis *p*-value to be smaller than 0.05. Then, power of a parametric approach like ShrinkBayes, which is essential in a multiple testing setting, is combined with the robustness of a nonparametric test. In this case, the median true FDR drops from 0.171 to 0.134 (target equals 0.1), while detecting 32 features on average instead of 37.4.

### Example: analysis of miRseq count data

#### ***Data***

We applied ShrinkBayes to a challenging data set. The data set contains miRseq counts of 2060 miRNAs (3p- and 5p-variants) for 55 resections from primary colon tumors (P) and corresponding metastases (M) coded by the covariate PM. In addition, several other covariates are available: indiv: most individuals correspond to 2 samples (one for P, M), but some have multiple measurements for M, because the metastasis occurred at multiple locations; organ: organ where the metastasis occurred; time: binary, indicating whether resections of the primary tumor and the metastasis were at different dates; chemo: binary, indicating whether chemotherapy was applied in between the resections. In addition to other software, ShrinkBayes provides two important extra features to correctly analyze these data: it explicitly accounts for excess of zeros and allows for random effects (here indiv). Both are important for appropriate inference. In addition, we demonstrate here that joint inference for related parameters like those corresponding to organ is feasible. Note that separate inference for each organ has limited power due to the small number of samples per organ. We focus on the statistical analysis. Preprocessing is described in the Additional file 1, Section 3, which also contains annotated R-code for the entire analysis, including inferences for organ and the P-M contrast.

#### ***Analysis***

The analysis consists of the following steps: 1) Likelihood specification for the counts, here the zero-inflated negative binomial one; 2a) Specification of the regression model. Here, the model  is the linear model with fixed effects PM, time, chemo and organ plus random effect indiv; 2b) Specification of the null-model ${ℳ}_{0}$: as , without organ; 3) Choice of parameters to shrink. Here, all fixed parameters plus the over-dispersion parameter of the negative binomial.

4) Estimation of priors for the purpose of shrinkage. Standard priors (Gaussian and inverse-Gamma) are used for all parameters, except for the inferential variable, organ, for which the multivariate mixture prior (5) is used; 5) Computation of posteriors under models  and ${ℳ}_{0}$, given the prior parameters; 6) Combination of the two posteriors to one given the parameters of the mixture prior; 7) Compute local and Bayesian false discovery rates (lfdr; BFDR). The most complex steps, 4) to 7), are completely automated including setting of tested defaults, which allows users with little experience in Bayesian computing to apply ShrinkBayes. The joint mixture prior is discussed above; other technical details are given in [2].

#### ***Discoveries***

**Figure 2:**
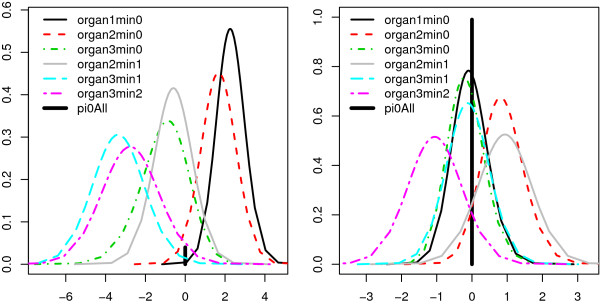
**Posterior densities and joint null-probability (pi0All) of 6 contrasts** ***β***_***i*** ***k ***_**- *β***_***i*** ***ℓ***_**, *k*** **>** ***ℓ*, representing log** ***e*-fold expression differences (x-axis) between 4 organs, for a significant miR (left) and non-significant miR (right).**

At BFDR = 0.10, we discovered 43 miRs for which organ is associated to expression in the metastasis. Figure 2 shows two posteriors of contrasts *β*_*ik *_- *β*_*i* *ℓ*_,*k *> *ℓ*, which help to explain differential or non-differential miR expression. For example, for the significant differential miR, which corresponds to the left display of Figure 2, differences are largest between organs 0 and 3 on one side and organs 1 and 2 on the other. To accommodate users, ShrinkBayes contains functions to easily produce such posterior plots and also summary tables. Importantly, the estimate of *p*_0 _in (5) is large, ${\hat{p}}_{0}=0.92$, which implies strong shrinkage of organ effects towards zero, rendering more  degrees of freedom’ and hence more power for other inferences. This is another strong aspect of ShrinkBayes: in studies with relatively few samples, multi-parameter shrinkage helps to increase power for a particular parameter of interest [4]. The idea of jointly shrinking multiple parameters was recently also adopted in [10], although their approach currently applies to *K*-group comparisons only.

## Discussion

For the choice of prior, we recommend to use the SpGG prior when inference on a parameter equalling zero is desired, because of its uniformly good performance in terms of FDR estimation and power. The SpNP prior is a good alternative which may be attractive in extremely small sample size settings for which the flexible shape of the non-parametric component is important (see also [4]). When using an interval null-hypothesis, *H*_0*i *_: |*β*_*i*_| < *δ*, inclusion of a spike is less relevant, so smooth (non-parametric) priors generally suffice.

Given the good performance of the SpGG prior in a univariate setting, it may be good to extend (5) to the multivariate analogue of the SpGG prior: a mixture of a multivariate point mass and a two-component Gaussian mixture product density. However, while this is conceptually feasible, it may be computationally cumbersome, because it would require combining several different fits from INLA under combinations of the components of the mixture.

Although ShrinkBayes is much more efficient that MCMC-based methods, it is computationally more demanding than frequentist counterparts like edgeR[8] and DESeq[9]. As an indication: the data example above (on approx 2,000 features) runs in approximately 30 minutes on 6 cpus of a Linux-cluster, whereas approximately 6 hours would be required for 100,000 features. For extremely large data sets, ShrinkBayes provides quick pre-screen functions, application of which potentially reduces computing time by a large factor.

We focused on sequencing count data for fairly complex designs. To our knowledge, extensively validated data are still not available for such studies, which hampers a thorough comparison between methods. Even when such a data set would be available, it is uncertain to what extent conclusions from one data set could be extrapolated to others, because the relative performance of a method may depend on many aspects such as the proportion of outliers and zero counts and/or the presence of multiple noise levels (e.g. within and between individuals). We emphasize that ShrinkBayes is currently the only RNAseq analysis method that can deal with the latter, by allowing random and mixed effects models, concepts that are widely accepted and used in other fields of statistical data analysis.

For simple designs, ShrinkBayes can be useful as well, in particular due to its good reproducibility, as shown for publicly available RNAseq data in [2]. ShrinkBayes also applies to Gaussian data, like mRNA microarray data or high-throughput RNA interference screens [4]. Use is similar, as illustrated in the ShrinkBayes R-vignette, which also contains additional examples on count data.

## Conclusion

We illustrated the versatility of ShrinkBayes on a data set which reflects a level of complexity that is common in clinical practice. With the decrease of costs for sequencing, we are likely to encounter such complex data sets frequently in the near future and ShrinkBayes provides the means and power to analyze these.

## Availability and requirements

**Project name: **ShrinkBayes **Project home page: **http://www.few.vu.nl/~mavdwiel/ShrinkBayes.html**Operating system: **Platform independent (developed on Linux)**Programming language: **R**Other requirements: **R (>= 3.0.1); R-packages: INLA, from http://www.r-inla.org and snowfall, VGAM, mclust, logcondens, Iso from CRAN**Licence: **GNU GPL

## Competing interests

The authors declare that they have no competing interests.

## Authors’ contributions

MAvdW developed the methodology and the software. MAvdW and MN wrote the manuscript. HMWV and TEB conceived of the miRseq study, which was carried out by MN. DS facilitated the sequencing and performed the initial analysis of the data. All authors read, discussed and approved the manuscript.

## Supplementary Material

Additional file 1 **Supplementary Material. **It contains: additional simulation results, a list of changes of the software with respect to previous versions, details on the Savage-Dickey approximation for marginal likelihood and extensive R-code for the miRseq example. Click here for file
