# Supplementary material for: ShrinkBayes: a versatile R-package for analysis of count-based sequencing data in complex study designs
Source: BMC Bioinformatics. 2014 Apr 26;15:116. doi: 10.1186/1471-2105-15-116 (PMC4098777; doi:10.1186/1471-2105-15-116)
Supplement: Additional file 1 — Supplementary Material. It contains: additional simulation results, a list of changes of the software with respect to previous versions, details on the Savage-Dickey approximation for marginal likelihood and extensive R-code for the miRseq example. [file 1471-2105-15-116-S1.pdf]

ShrinkBayes: a versatile R-package for analysis of count-based sequencing data in complex study designs

Supplementary Material

Mark A. van de Wiel[1,4]*, Maarten Neerincx[2], Tineke E. Buffart[2], Daoud Sie[3], Henk M.W. Verheul[2]
[1]Department of Epidemiology and Biostatistics, [2]Department of Medical Oncology and [3]Department of Pathology, VU University Medical Center, Amsterdam, The Netherlands
[4]Department of Mathematics, VU University, Amsterdam, The Netherlands.

This document provides Supplementary Material for the paper "ShrinkBayes: a versatile R-package for analysis of count-based sequencing data in complex study designs".

1 Simulation results for FDR estimation

1.1 Evaluating priors for various effect size distributions

To study the inferential performance of ShrinkBayes when the null-hypotheses are of the equality-type, $H_{0i} : \beta_i = 0$, we compared 4 types of spike-containing priors under 20 different settings. These settings are defined by all combinations of: sample sizes, $N = 2 * n = 10, 20$; proportion of true null-hypotheses, $p_0 = 0.8, 0.95$; and 5 distributions of the effect size for the non-zero effects in a two-sample study: $\mathrm{Gamma}(1,1), \mathrm{t}(4)$, $\mathrm{Uniform}[-1.5, 1.5]$, $\mathrm{N}(0, (0.75)^2)$, $0.5\mathrm{N}(-0.4, (0.4)^2) + 0.5\mathrm{N}(0.4, (0.4)^2)$. We focussed on small sample sizes, because for those the relative effect of the prior is likely to be most prominent. The four priors are:

$$
\begin{aligned}
\mathrm{SpG} &= p_0\delta_0 + (1 - p_0)N(0, \sigma^2) && \text{'Spike-Gauss'}\\
\mathrm{SpGG} &= p_0\delta_0 + p_{-1}N(-\mu, \tau^2) + p_1 N(\mu, \tau^2) && \text{'Spike-Gauss-Gauss'}\\
\mathrm{SpSlab} &= p_0\delta_0 + (1 - p_0)N(0, 0.1^2) && \text{'Spike-Slab'}\\
\mathrm{SpNP} &= p_0\delta_0 + (1 - p_0)F_{\mathrm{NP}} && \text{'Spike-Nonparametric'},
\end{aligned}
$$

where δ_0 is the dirac delta function. All parameters are determined by explicitly maximizing the total (log-)marginal likelihood, where F_{NP} is obtained by the iterative marginal procedure (Van de Wiel et al., 2012) with the restriction that it contains maximally one mode on both the negative and positive half-plane. This helps to identify F_{NP} together with p_0.

The priors were evaluated using the following 4 criteria:

1. Accuracy of FDR estimation
2. Number of detections at given (B)FDR cut-off
3. Area-under-the-curve (AUC)
4. The root-mean-square-error for true non-null features: $\mathrm{RMSE} = \sqrt{\sum_{i\in\mathcal{H}_1}(\hat{\beta}_i - \beta_i)^2/|\mathcal{H}_1|}$, where $\mathcal{H}_1$ is the set of non-null features.

*mark.vdwiel@vumc.nl

The first 3 criteria focus on inference performance, the last one on estimation.

Table 1 displays the results for FDR-estimation at target FDR=0.1. SpGG generally performs well across all distributions; SpG and SpNP are close, but somewhat inferior for the Gamma-distribution and (not surprisingly) the Gaussian mixture. SpSlab is a conservative alternative, which is not surprising given the wide slab, which represents the non-null features.

Table 2 shows the number of detections for estimate $\text{BFDR}(t, Y) \approx 0.1$. From this table we conclude that the conservative behavior of SpSlab comes at a price: the number of detections is much lower than for the other 3 methods.

Area-under-the-curves (AUC) were computed for the ROC-curves, see Table 3. Results are fairly similar for most distributions, indicating that the ranking of features (which determines the ROC-curve) does not strongly depend on the prior. SpGG seems to outperform the other three for the Gamma-distribution. Note that we also computed AUC for partial ROC-curves, restricted to false positive rate ≤ 0.2. Since results are in the same spirit as those presented here, we do not show these.

Finally, results on accuracies of the estimations are given in Table 4. Here, one may expect SpNP to perform best, given the flexibility of the smooth non-parametric component. We observe, however, that SpGG is a very good competitor which indicates that the mixture is flexible enough for estimation purposes.

1.2 The case $p_0 = 1$

We also evaluated the performances of the four priors in case the proportion of true null-hypotheses, p_0, equals 1. Then, of course, the estimate of p_0 should be close to 1. Also, the (B)FDR estimate, $\text{BFDR}(t, Y)$, for threshold t equal to the smallest posterior null-probability $P(H_{0i}|Y), i = 1, \ldots, p$ should be high, so that the procedure will not declare significant findings at any reasonable threshold. Table 5 displays the results for $N = 10, 20$. Although none of the priors renders any significant result at $\text{BFDR} < 0.1$, we observe that the results are best for the SpGG prior.

2 Changes with respect to `ShrinkBayes` version 1.6.

2.1 List of changes

`ShrinkBayes` versions 2.3 and higher contain several additional functionalities and changes with respect to `ShrinkBayes` version 1.6, which corresponds to Van de Wiel et al. (2012):

(a) Inference for nested models that differ by more than one variable is now feasible.

(b) Spike-and-Slab priors are included.

(c) Spike-and-nonparametric priors are included.

(d) Parametric mixture priors are allowed to contain non-equal mixture proportions for negative and positive effects.

(e) Use of mixture priors is easier due to automatic refinement of parameter search.

(f) Comparison of full model $\mathcal{M}$ and null-model $\mathcal{M}_0$ can now be performed in two ways. Either exact (recommended), by computing a Bayes Factor from fits under both models or by applying the Savage-Dickey approximation for the Bayes Factor (see below). The latter

is computationally faster, because it requires to fit model $\mathcal{M}$ only. In addition, it is very convenient for contrasts for which a null-model can not always be defined without the use of constraints. However, it is somewhat less accurate sometimes, because it may depend on parametrization (Wetzels et al., 2010).

(g) Gaussian approximation to the marginal likelihood is used rather than the integrated version (Rue et al., 2009), because the former renders better performance for comparing a null-model with a full model.

(h) Convergence is assessed by total marginal likelihood: if the change is less than 0.1% with respect to the previous value iteration is stopped. This is faster than assessing convergence by Kolmogorov-Smirnov distances per parameter. In addition, it prevents 'walking on a ridge' of the likelihood landscape. The latter is particularly important for p_0 estimation.

(i) Default for the number of decimal digits has changed. More digits were needed for accurate marginal likelihood computation.

(j) Function `SummaryTable` is added, which supplies summary statistics (lfdr, BFDR, posterior mean) for (significant) features in a data frame format.

(k) Function `plotPoster` is added, which plots posterior densities.

2.2 Savage-Dickey approximation

The Savage-Dickey approximation of the Bayes Factor, which equals $\mathrm{BF} = \mathrm{ML}(Y;\mathcal{M}_0)/\mathrm{ML}(Y;\mathcal{M})$: the ratio of marginal likelihoods, is obtained by a conditioning argument (assuming null model $\mathcal{M}_0$ is the same as full model $\mathcal{M}$, except for excluding parameter β):

$$\mathrm{ML}(Y;\mathcal{M}_0) \approx \mathrm{ML}(Y;\mathcal{M}|\beta = 0) = f_{\mathcal{M}}(0|Y)f_{\mathcal{M}}(Y)/f(0) = \frac{f_{\mathcal{M}}(0|Y)}{f(0)}\mathrm{ML}(Y;\mathcal{M}),$$

so Bayes Factor $\mathrm{BF} \approx \frac{f_{\mathcal{M}}(0|Y)}{f(0)}$, which is the ratio of the posterior and prior density on 0. Accuracy of the approximation depends on the validity of the conditioning argument (Wetzels et al., 2010).

3 Analysis of miRseq colon tumor plus metastasis data

3.1 Preprocessing of miRseq colon tumor plus metastasis data

The miRseq data consist of 2060 features representing 3p- and 5p-variants of novel and known human microRNAs for 55 resections. We did not apply any count-based filtering, because this may introduce biases in a multiple testing setting (van Iterson et al., 2010) and `ShrinkBayes` is able to process features with many low counts. We normalized the counts using edgeR's function `calcNormFactors`, with `method = "TMM"` (Oshlack et al., 2010).

3.2 R code

The code below is also available on http://www.few.vu.nl/˜mavdwiel/ShrinkBayes.html, as a "ready-to-run" R-script.

First, we load the library, the miRseq data and the design, then we set the number of cores to be used for parallel computing.

```
library(ShrinkBayes)
data(mirseqnorm)
data(designmirseq)
designmirseq
nc <- 6
```

Then, retrieve the covariates:

```
PM <- designmirseq$PM
indiv <- designmirseq$indiv
timepos <- designmirseq$timepos
chemo <- designmirseq$chemo
organ1 <- designmirseq$organ1
organ2 <- designmirseq$organ2
organ3 <- designmirseq$organ3
```

Specify the full model $\mathcal{M}$ and null-model $\mathcal{M}_0$. INLA notation f() is used for random effects.

```
form = y ~ 1 + PM + timepos + chemo + organ1 + organ2 + organ3 + f(indiv)
form0 = y ~ 1 + PM + timepos + chemo + f(indiv)
```

Start simultaneous shrinkage for parameters specified under `shrinkfixed` (contains parameter of primary interest, here `PM`) and under `shrinkaddfixed`; `excludefornull` specifies effects left out in the null-model (`form0`) for the purpose of multi-parameter inference. Default likelihood is zero-inflated negative binomial.

```
shrinksimul <- ShrinkSeq(form, mirseqnorm, shrinkfixed="PM",
+ shrinkaddfixed=c("organ1","organ2","organ3","chemo","timepos"),
+ excludefornull=c("organ1","organ2","organ3"), ncpus = nc)
```

Define all contrasts between organs 1 to 3.

```
lcv <- AllComp(c("organ1","organ2","organ3"))
```

Fit models $\mathcal{M}$ and $\mathcal{M}_0$ for all features. The contrasts are only relevant under model $\mathcal{M}$. We set `finalprior=TRUE`, because the priors resulting from the `ShrinkSeq` are the final ones (we do not intend to update these; see remarks at the end of this section).

```
fitall <- FitAllShrink(form, mirseqnorm, shrinksimul, finalprior=TRUE,
+ ncpus = nc,  lincomb=lcv)
fitall0 <- FitAllShrink(form0, mirseqnorm, shrinksimul, finalprior=TRUE,
+ ncpus = nc)
```

Compute posteriors by combining the posteriors stored in `fitall` and `fitall0`. Posteriors are computed for all 6 contrasts, represented by parameters `c("organ1","organ2","organ3")` (contrast with respect to baseline organ 0) and the contrast defined in object lcv.

```
postercombined <- BFUpdatePosterior(fitall, updateoutput=shrinksimul, fitall0,
+ shrinkpara=c("organ1","organ2","organ3"), shrinklc=names(lcv))
```

Compute summary table for all miRNAs with significant effect of 'organ' at BFDR ≤ 0.10. See remarks below on `direction`.

```
ST <- SummaryTable(postercombined, direction="equal")
```

Plot posteriors for (significant) miRNA 539, including posterior null-probability (= local fdr).

```
plotPoster(539,postercombined)
```

Further remarks on the code

The precision parameter of `indiv` is not shrunken, because a) the non-shrunken estimate is likely to be accurate given the number of individuals (26) in the study and b) modeling `indiv` as a random effect already shrinks the effect of each individual.

When one aims to further update the priors (using either of the functions `NonparaUpdatePrior` or `MixtureUpdatePrior`, see example in next section) it is better to disperse the priors returned by `ShrinkSeq` when using FitAllShrink (for numerical stability: Van de Wiel et al., 2012). This is achieved by using the default `finalprior=FALSE`. We built in an extra safeguard in the function `BFUpdatePosterior`: it will not run when the prior was (mistakenly) dispersed, so when `finalprior=FALSE` was used.

About `direction="equal"` in the function `SummaryTable`: in a multi-parameter setting this tests the joint null-hypothesis of all parameters equalling zero. Alternatively, one may opt to use `direction="two-sided"`. In such a case, a multiple comparisons approach is taken (see Van de Wiel et al., 2012): a BFDR is computed for all parameters (and/or contrasts), and a feature is selected if the minimum BFDR ≤ 0.10. The latter approach is slightly more conservative (e.g. for this example, 37 instead of 43 miRs would be selected).

3.3 Inference for (organ-specific) P-M contrast

Load the data and the design, set the number of cores, and retrieve the covariates as in the previous section. Since the overall P-M effect should reflect the difference of P with respect to M on average across organs, we redefine the effect of the baseline organ 0 to be minus the sum of the effects of the other organs (which implies a sum-to-zero constraint for the organ effects).

```
whredefine <- which(PM=="M" & organ1==0 & organ2==0 & organ3==0)
organ1a <- organ1; organ1a[whredefine] <- -1
organ2a <- organ2; organ2a[whredefine] <- -1
organ3a <- organ3; organ3a[whredefine] <- -1
```

Specify the full model $\mathcal{M}$ and null-model $\mathcal{M}_0$. INLA notation f() is used for random effects.

```
form = y ~ 1 + PM + timepos + chemo + organ1a + organ2a + organ3a + f(indiv)
form0 = y ~ 1 + organ1a + organ2a + organ3a + timepos + chemo + f(indiv)
```

We are also interested in the organ-specific P-M contrast for organ 0. This contrast subtracts the coefficients for organ0 (-1,-1,-1) from the coefficient for P. Note that for a factor variable (like `PM`) the level should be pasted to the name of the factor: `PM`, level P becomes `PMP`.

```
lc <- inla.make.lincomb(PMP = 1, organ1a=1, organ2a=1, organ3a=1)
names(lc) <- "PminMorgan0"
```

Start simultaneous shrinkage for parameters specified under `shrinkfixed` and under `shrinkaddfixed`. Note that for inference on one parameter (as we intend here), we first simply obtain smooth parametric priors, so option `excludefornull` does not have to be used here.

```
shrinksimul <- ShrinkSeq(form, mirseqnorm, shrinkfixed="PM",
+ shrinkaddfixed=c("organ1a","organ2a","organ3a","chemo","timepos"), ncpus = nc)
```

Fit models $\mathcal{M}$ and $\mathcal{M}_0$ for all features. Because we also want to compute posteriors for the organ-specific contrast `lc`, we need to specify `lincomb=lc` for the full model.

```
fitall <- FitAllShrink(form, mirseqnorm, shrinksimul, ncpus = nc, lincomb=lc)
fitall0 <- FitAllShrink(form0, mirseqnorm, shrinksimul, ncpus = nc)
```

Update the prior for the P-M contrast, which is coded by parameter `PM`, to a Spike-Gaussian-Gaussian (SpGG) prior and recompute posteriors using the SpGG prior.

```
mixtprior <- MixtureUpdatePrior(fitall, fitall0, shrinkpara="PM", ncpus = nc)
mixtpostshr <- MixtureUpdatePosterior(fitall, mixtprior, fitall0, ncpus = nc)
```

Compute summary table for *all* miRNAs, by setting BFDR threshold = 1. For uni-parameter inference we advise to use the default `direction="two-sided"`, which combines two one-sided hypotheses, rather than `direction="equal"`. The former is more appropriate when the posterior is very wide and spanning 0.

```
ST <- SummaryTable(mixtpostshr, BFDRthr=1)
```

Plot posterior for miRNA 333, including posterior null-probability.

```
plotPoster(333, mixtpostshr, plotlabel="P-M")
```

The inference for the overall P-M contrast does not render significant miRNAs at BFDR = 0.1. The small sample sizes for organs 1 to 3 (5,3, and 5, respectively) and the differences between the four organs most likely cause this. We now focus on the contrast between P and organ 0 (sample size 15). For this we can conveniently directly use the `fitall` object, so it is not necessary to also fit a new null-model: the Bayes factor of the full model and the null model is computed by the Savage-Dickey approximation (see Section 2.1). Hence, we immediately compute a mixture prior for the contrast of interest, where `fitall0=NULL` by default, and update the posteriors:

```
mixtprior0 <- MixtureUpdatePrior(fitall, shrinklc="PminMorgan0", ncpus = nc)
mixtpostshr0 <- MixtureUpdatePosterior(fitall, mixtprior0, ncpus = nc)
```

Again, we compute a summary table, with BFDR threshold 0.1:

```
ST0<- SummaryTable(mixtpostshr0)
```

This produces 12 significant miRs. We plot miR 223.

```
plotPoster(223, mixtpostshr0, plotlabel="P-MOrgan0")
```

3.4 Computing time

Computing time depends on the number of samples, number of features, complexity of the design and the number of cpus available for parallel computing. The examples above both run in approximately 30 min. on 6 cpus of a Linux-cluster. The functions `ShrinkSeq`, `MixtureUpdatePrior` and `FitAllShrink` demand most of the time, but only the latter one takes more time with more features. E.g. for 100,000 features computing time would be approximately 500 min. Extremely large data sets can first be screened with p-value-based methods using `ShrinkBayes` functions `FastScreenP` and `ScreenData`, which potentially reduces computing time by a large factor.

4 Tables

Distr	N	p_0	SpG	SpGG	SpSlab	SpNP
Gamma	10	0.80	0.114	0.114	0.070	0.132
Gamma	20	0.80	0.062	0.089	0.044	0.091
Gamma	10	0.95	0.078	0.093	0.078	0.062
Gamma	20	0.95	0.081	0.096	0.032	0.14
t	10	0.80	0.113	0.123	0.079	0.121
t	20	0.80	0.096	0.092	0.047	0.101
t	10	0.95	0.131	0.115	0.096	0.115
t	20	0.95	0.086	0.094	0.047	0.141
Uniform	10	0.80	0.126	0.131	0.143	0.127
Uniform	20	0.80	0.097	0.095	0.025	0.083
Uniform	10	0.95	NA	NA	NA	NA
Uniform	20	0.95	0.081	0.091	0.066	0.105
Gauss	10	0.80	0.104	0.121	0.158	0.104
Gauss	20	0.80	0.104	0.1	0.015	0.081
Gauss	10	0.95	NA	NA	NA	NA
Gauss	20	0.95	0.107	0.108	0.067	0.129
MixtGauss	10	0.80	NA	NA	NA	NA
MixtGauss	20	0.80	0.092	0.106	0	0.103
MixtGauss	10	0.95	NA	NA	NA	NA
MixtGauss	20	0.95	0.179	0.12	0.167	0.185

Table 1: True FDRs for target FDR = 0.10, estimated by BFDR. NA corresponds to settings with less than 5 detections.

Distr	N	p_0	SpG	SpGG	SpSlab	SpNP
Gamma	10	0.80	491	614	399	676
Gamma	20	0.80	805	954	723	1009
Gamma	10	0.95	77	108	77	112
Gamma	20	0.95	185	208	156	228
t	10	0.80	488	537	404	555
t	20	0.80	915	899	726	923
t	10	0.95	61	61	52	61
t	20	0.95	162	160	129	177
Uniform	10	0.80	127	145	28	142
Uniform	20	0.80	710	706	365	661
Uniform	10	0.95	5	0	2	0
Uniform	20	0.95	124	132	61	124
Gauss	10	0.80	48	58	19	48
Gauss	20	0.80	402	401	197	372
Gauss	10	0.95	1	0	1	0
Gauss	20	0.95	75	74	45	70
MixtGauss	10	0.80	0	0	0	4
MixtGauss	20	0.80	184	199	37	203
MixtGauss	10	0.95	0	0	1	0
MixtGauss	20	0.95	28	25	6	27

Table 2: Number of detections at $\mathrm{BFDR}(t, Y) \approx 0.1$

Distr	N	p_0	SpG	SpGG	SpSlab	SpNP
Gamma	10	0.80	0.742	0.771	0.741	0.786
Gamma	20	0.80	0.803	0.834	0.802	0.844
Gamma	10	0.95	0.759	0.806	0.756	0.750
Gamma	20	0.95	0.817	0.841	0.815	0.813
t	10	0.80	0.766	0.766	0.767	0.764
t	20	0.80	0.805	0.805	0.805	0.805
t	10	0.95	0.752	0.752	0.751	0.749
t	20	0.95	0.810	0.810	0.810	0.809
Uniform	10	0.80	0.731	0.733	0.732	0.731
Uniform	20	0.80	0.796	0.799	0.798	0.798
Uniform	10	0.95	0.745	0.741	0.746	0.742
Uniform	20	0.95	0.833	0.833	0.832	0.829
Gauss	10	0.80	0.682	0.683	0.681	0.681
Gauss	20	0.80	0.746	0.746	0.747	0.746
Gauss	10	0.95	0.695	0.692	0.694	0.692
Gauss	20	0.95	0.764	0.764	0.762	0.762
MixtGauss	10	0.80	0.638	0.639	0.637	0.637
MixtGauss	20	0.80	0.695	0.696	0.695	0.696
MixtGauss	10	0.95	0.632	0.630	0.631	0.629
MixtGauss	20	0.95	0.721	0.720	0.723	0.721

Table 3: Area-under-the-curves (AUC)

Distr	N	p_0	SpG	SpGG	SpSlab	SpNP
Gamma	10	0.80	0.618	0.564	0.668	0.540
Gamma	20	0.80	0.462	0.420	0.490	0.406
Gamma	10	0.95	0.823	0.718	0.829	0.703
Gamma	20	0.95	0.520	0.491	0.548	0.466
t	10	0.80	0.726	0.722	0.714	0.708
t	20	0.80	0.651	0.650	0.529	0.644
t	10	0.95	0.830	0.819	0.843	0.835
t	20	0.95	0.579	0.582	0.634	0.599
Uniform	10	0.80	0.594	0.573	0.757	0.592
Uniform	20	0.80	0.456	0.450	0.588	0.461
Uniform	10	0.95	0.739	0.737	0.854	0.718
Uniform	20	0.95	0.529	0.526	0.650	0.535
Gauss	10	0.80	0.553	0.557	0.670	0.561
Gauss	20	0.80	0.430	0.431	0.536	0.441
Gauss	10	0.95	0.673	0.656	0.739	0.652
Gauss	20	0.95	0.500	0.499	0.585	0.503
MixtGauss	10	0.80	0.472	0.460	0.548	0.458
MixtGauss	20	0.80	0.398	0.392	0.502	0.390
MixtGauss	10	0.95	0.576	0.561	0.600	0.545
MixtGauss	20	0.95	0.442	0.449	0.514	0.439

Table 4: RMSE for non-zero features

N		SpG	SpGG	SpSlab	SpNP
10	$\hat{p}_0$	1.000	1.000	1.000	0.949
20	$\hat{p}_0$	0.985	0.998	1.000	0.956
10	min. BFDR	1.000	1.000	0.289	0.448
20	min. BFDR	0.481	0.926	0.992	0.204

Table 5: Estimates of p_0 and minimum BFDR

References

Oshlack, A. et al. (2010). From RNA-seq reads to differential expression results. *Genome Biol.*, **11**, 220.

Rue, H. et al. (2009). Approximate Bayesian inference for latent Gaussian models by using integrated nested Laplace approximations (with discussion). *J. R. Statist. Soc. B*, **71**, 319–392.

Van de Wiel, M.A. et al. (2012). Bayesian analysis of RNA sequencing data by estimating multiple shrinkage priors. *Biostatistics*, **14**, 113–128.

van Iterson, M. et al. (2010). Filtering, FDR and power. *BMC Bioinformatics*, **11**, 450.

Wetzels, R. et al. (2010). An encompassing prior generalization of the Savage-Dickey density ratio. *Comp. Stat. Data Anal.*, **54**, 2094–2102.
